# Notch Promotes Neural Lineage Entry by Pluripotent Embryonic Stem Cells

**DOI:** 10.1371/journal.pbio.0040121

**Published:** 2006-04-11

**Authors:** Sally Lowell, Alexandra Benchoua, Barry Heavey, Austin G Smith

**Affiliations:** **1**Centre Development in Stem Cell Biology, Institute for Stem Cell Research, School of Biological Sciences, University of Edinburgh, Edinburgh, United Kingdom; **2**INSERM U 421/I-STEM, Faculté de Médecine, Evry-Cedex, France; **3**Institute for Stem Cell Biology, University of Cambridge, Cambridge, United Kingdom; National Institute for Medical ResearchUnited Kingdom

## Abstract

A central challenge in embryonic stem (ES) cell biology is to understand how to impose direction on primary lineage commitment. In basal culture conditions, the majority of ES cells convert asynchronously into neural cells. However, many cells resist differentiation and others adopt nonneural fates. Mosaic activation of the neural reporter *Sox-*green fluorescent protein suggests regulation by cell-cell interactions. We detected expression of Notch receptors and ligands in mouse ES cells and investigated the role of this pathway. Genetic manipulation to activate Notch constitutively does not alter the stem cell phenotype. However, upon withdrawal of self-renewal stimuli, differentiation is directed rapidly and exclusively into the neural lineage. Conversely, pharmacological or genetic interference with Notch signalling suppresses the neural fate choice. Notch promotion of neural commitment requires parallel signalling through the fibroblast growth factor receptor. Stromal cells expressing Notch ligand stimulate neural specification of human ES cells, indicating that this is a conserved pathway in pluripotent stem cells. These findings define an unexpected and decisive role for Notch in ES cell fate determination. Limiting activation of endogenous Notch results in heterogeneous lineage commitment. Manipulation of Notch signalling is therefore likely to be a key factor in taking command of ES cell lineage choice.

## Abstract 2

Genetic manipulations reveal a novel role of Notch signaling in promoting and directing embryonic stem cells toward neural fates and suppressing differentiation into other lineages.

## Introduction

Embryonic stem (ES) cells can generate all somatic cell types. They constitute a unique cellular system for uncovering the molecular basis of pluripotency and delineating mechanisms of primary lineage commitment. ES cells also present opportunities for disease modelling, pharmacological screening, and cell based therapies. A key challenge is to understand the cues that direct ES cells into particular developmental pathways. Conventional embryoid body differentiation protocols generate a chaotic mixture of differentiated cell types [ 1]. Selective regimes can be used to enrich for cell types of interest [ 2, 3], but the initial lineage commitment process remains obscure and uncontrolled.

Conditions have been established for monolayer differentiation of ES cells [ 4– 6], reducing the complexity compared with multicellular aggregation or coculture systems. On withdrawal of self-renewal stimuli, ES cells will readily generate neural progenitors in adherent monoculture. Neural commitment requires the absence of exogenous serum factors or bone morphogenetic proteins (BMPs), which act as potent antineural factors, and appears to be driven by autocrine signals, including fibroblast growth factors [ 7]. However, even under these simple culture conditions in which all cells are in a similar environment, 30% to 40% of cells resist neural specification. Of these, around half differentiate into nonneural lineages, while the others remain as ES cells. The failure of the latter cells to differentiate into the neural lineage is not due to an intrinsic lack of neural potential: they are often able to differentiate subsequently, either within the same culture or after replating. This implies either that a neural inductive signal is in limiting supply, or that antineural signals are prevalent. Furthermore, newly born neural cells, visualised using the vital reporter of neural specification *Sox1*GFP [ 8], are closely interspersed with undifferentiated cells in a salt-and-pepper pattern (see below), indicating intraclonal rather than interclonal variation. These observations suggest that local cell-cell interactions regulate neural specification.

Notch signalling is used in many different tissues to regulate differentiation decisions by mediating signalling between adjacent cells [ 9]. This pathway is classically deployed to restrict the spread of cell differentiation, a process called lateral inhibition [ 10]. In other contexts, however, Notch promotes neighbouring cells to adopt the same fate, lateral induction [ 11]. In this study we report that Notch receptors and ligands are expressed in ES cells where they play a previously unsuspected role in the promotion of primary neural fate.

## Results

### Undifferentiated ES Cells Express Notch Receptors and Ligands

**Figure 1:**
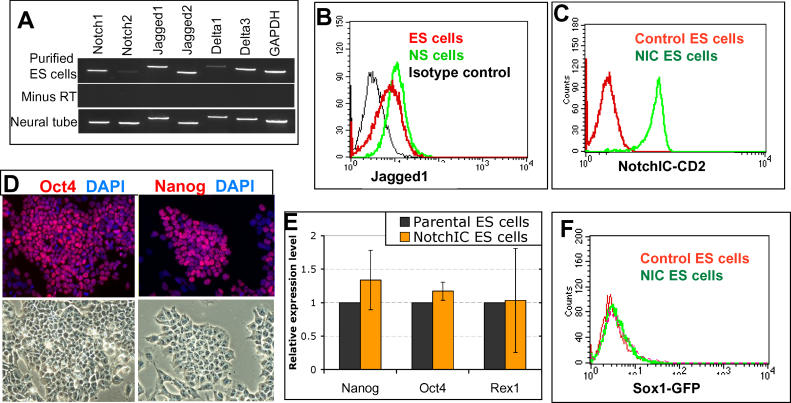
R26NotchIC Does Not Impair ES Cell Self-Renewal (A) RT-PCR for Notch receptors and ligands in Oct4-GiP ES cells selected in puromycin (“purified ES cells”). E13.5 neural tissue was used as a positive control. (B) FACS analysis of Jagged1 expression in live unpermeabilised 46C ES cells using single-chain antibody to Jagged1. NS cells [ 42] were used as a positive control and a single-chain antibody to the irrelevant intracellular antigen Desmin was used for the negative control. (C) FACS analysis of CD2 expression in R26NotchIC cells with parental 46C ES cells as controls. (D–F) Analysis of R26NotchIC ES cells or parental control 46C ES cells after 10 to 12 passages in LIF+ serum. (D) R26NotchIC ES cells under phase contrast or stained for ES cell markers as indicated (average of three independent samples). (E) Quantitative RT-PCR analysis of ES cell markers, normalised to GAPDH, and displayed relative to expression in 46C ES cells. (F) FACS analysis of *Sox1*GFP expression.

To assess a potential contribution of the Notch pathway to ES cell fate decisions, we surveyed expression of Notch receptors and ligands in undifferentiated mouse ES cells. To this end, we made use of Oct4-GiP ES cells in which a puromycin resistance gene is expressed under the control of the pluripotent cell-specific Oct4 promoter, such that selection in puromycin eliminates differentiated cells [ 12, 13]. Notch1 and its ligands, Jagged1, Jagged2, and Delta3, are readily detected by RT-PCR on total RNA prepared from purified Oct4-GiP ES cells ( Figure 1A). By fluorescence activated cell sorter (FACS) analysis using a single-chain Fv antibody, we found that around half of undifferentiated ES cells express Jagged1 ( Figure 1B).

### Constitutive Expression of NotchIC in *Sox1* Reporter ES Cells

Notch signal transduction is mediated via proteolytic cleavages at the cell membrane upon ligand binding. The end effect is to release the Notch intracellular domain (NotchIC), which translocates to the nucleus and binds to the RBPJk protein (also known as CSL), converting it from a transcriptional repressor to an activator [ 9]. The requirement for ligand can by bypassed by expression of recombinant NotchIC [ 14]. We introduced NotchIC into ES cells via episomal transduction using the high-expression pPyCAGIP vector [ 15]. Transfected cells exhibited a massive increase in Notch activity (1,600 ± 500-fold increase according to a 12xRBPJk-luciferase reporter assay). They stopped dividing, flattened and spread over the culture surface, and did not survive beyond a few days (unpublished data). This suggests that very high expression of NotchIC is toxic, although it is also possible that it induces differentiation into a cell type that cannot survive under these culture conditions. We therefore devised a strategy for achieving moderate expression of NotchIC, based on targeting into the *ROSA26* locus [ 16], which drives ubiquitous but relatively moderate expression. We preceded the NotchIC sequence with a floxed transcriptional termination sequence and PGKneo cassette, such that expression was dependent upon Cre recombinase (CRE) activation. IRES-human CD2 was appended 3′ to the NotchIC. Human CD2 is a cell surface molecule with no apparent phenotypic effect on mouse cells, used here as a tag to indicate NotchIC expression. Targeting was carried out in 46C ES cells that harbour a knock-in of GFP to one allele of the neural specification marker gene *Sox1* [ 8] and thus act as a convenient experimental system for monitoring neural induction [ 7, 17]. A clonal line of NotchIC-targeted ES cells was transfected with CRE to excise the termination sequence and PGKneo, thereby activating constitutive transcription of NotchIC-IRES-CD2 under control of ROSA26 regulatory elements. The successfully deleted population, designated “R26NotchIC cells,” was separated from the undeleted population by FACS based on CD2 expression ( Figure 1C). The undeleted population and parental 46C cells were used as independent control populations and behaved similarly in all experiments. A second independently targeted and excised clone yielded similar results to those described below.

To confirm that NotchIC is expressed and functional in the R26NotchIC cells, we used the 12xRBPJk-luciferase assay. The relative increase in Notch activity in R26NotchIC cells compared with control populations was on average 6 ± 2-fold when cells were maintained in the presence of leukemia inhibitory factor (LIF) and serum.

Under self-renewal culture conditions in the presence of LIF and serum or BMP4 [ 18, 19], R26NotchIC ES cells show no overt difference in growth rate or undifferentiated morphology compared to either undeleted or parental 46C ES cells. Population doubling times were similar over more than 20 passages (unpublished data), and R26NotchIC cells express pluripotency markers such as Oct4 and Nanog [ 20], and lack markers of differentiation ( Figure 1D– 1F and unpublished data). They have a modal average of 40 chromosomes in metaphase spreads. We conclude that moderate levels of activated Notch do not impair ES cell self-renewal.

### R26NotchIC Cells Undergo Accelerated and High-Frequency Neural Specification

**Figure 2:**
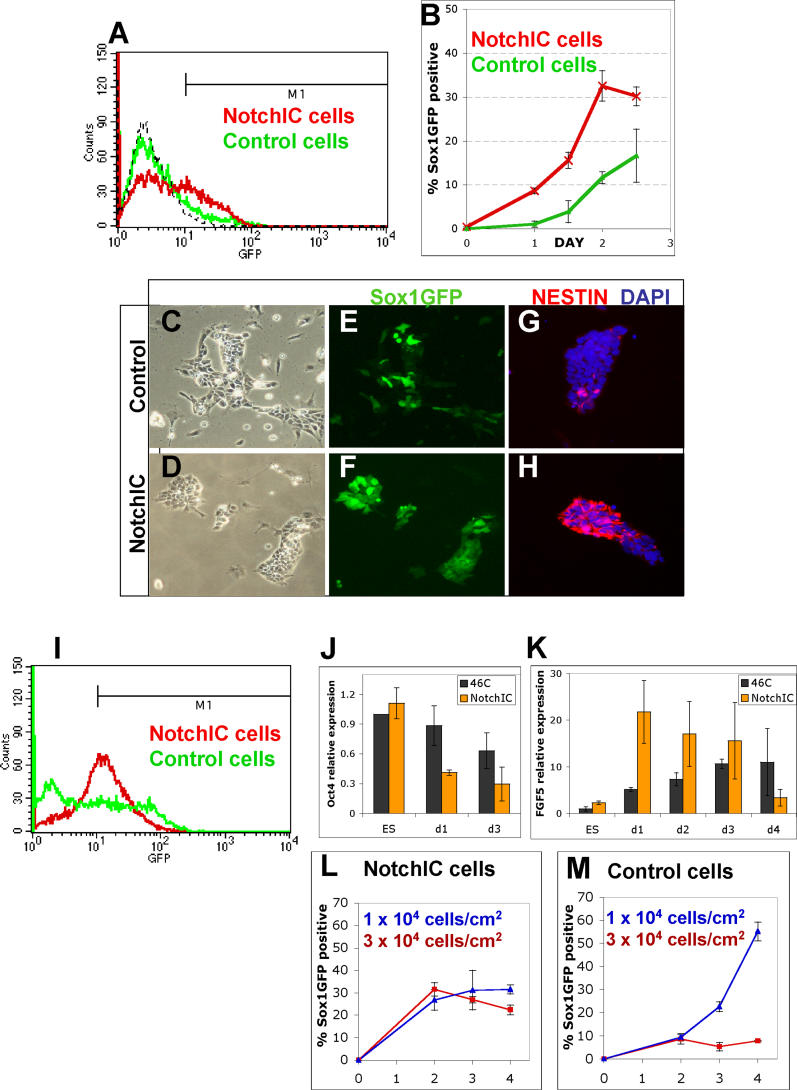
R26NotchIC Cells Undergo Accelerated Neural Specification R26NotchIC ES cells or parental control 46C cells cultured under monolayer differentiation conditions. (A) Typical FACS profile of *Sox1*GFP expression after 48 h. *Sox1*GFP+ cells were scored using gate M1. (B) Proportion of *Sox1*GFP+ cells at various time points (average of triplicates). (C–H) Intact cultures at 72 h of monolayer differentiation, shown in phase contrast or stained for markers as indicated. (I) Typical FACS profile of *Sox1*GFP expression after 5 d. M1 is the gate used throughout to quantify the proportion *Sox1*GFP+ cells: note that this FACS profile indicates a more striking difference between control and NotchIC populations than is reflected by our conservative quantification. (J and K) Quantitative RT-PCR for Oct4 and for FGF5 during monolayer differentiation of R26NotchIC cells (NotchIC) or control parental 46C cells. Levels were normalised to GAPDH and are displayed relative to expression in 46C ES cells. (L and M) FACS analysis of the proportion of *Sox1*GFP+ cells for R26-NotchIC cells or parental 46C cells at various time points from triplicate cultures at normal density (10 ^4^ cells/cm ^2^) or higher density (3 × 10 ^4^ cells/cm ^2^) (average of triplicates).

We then examined the response of R26NotchIC ES cells to withdrawal of self-renewal stimuli. We transferred NotchIC or control ES cells into a differentiation induction regime comprising adherent monolayer culture in the absence of exogenous growth factors (described in detail in [ 21]). These conditions are permissive for neural commitment driven by autocrine signalling [ 7]. Interestingly, we found that Notch activity, measured by the RBPJk-luciferase reporter, increased almost 4-fold under these conditions. Cells were harvested and analysed by flow cytometry for *Sox1*GFP expression every 12 h. As previously described [ 7], control ES cells generate GFP+ cells progressively after an initial lag: fewer than 2% could be detected after 24 h and only 11 ± 1% after 48 h. In contrast, NotchIC cultures ( Figure 2) contained 9 ± 1% *Sox1*GFP+ cells at 24 h, increasing to 32 ± 3% by the second day ( Figure 2A and 2B). In an alternative neural differentiation protocol, coculture on stromal PA6 cells [ 22], R26NotchIC cells also showed accelerated acquisition of *Sox1*GFP expression compared with control cells (unpublished data).

The distribution of *Sox1*GFP+ cells during monolayer differentiation was monitored by fluorescence microscopy. In control ES cell cultures, GFP-positive cells initially appear randomly interspersed with GFP-negative cells in a “salt and pepper” pattern ( Figure 2C and 2E). In contrast, in NotchIC cultures, GFP+ cells appear as coherent groups ( Figure 2D and 2F). To corroborate that *Sox1*GFP marked entry into the neural lineage, we immunostained cultures for the early neural marker, nestin, and found that this was expressed in a similar pattern to sox1, although it tended to appear approximately 12 to 24 h later than the first GFP+ cells ( Figure 2G and 2H). We also noted a difference in the variability of GFP intensity between cells: control cultures contain a mixture of bright and less intensely GFP positive cells ( Figure 2I [Control cells] and [ 8]), whereas NotchIC cells show uniformly moderate GFP expression ( Figure 2I [NotchIC cells]).

A direct effect of Notch at the onset of ES cell differentiation is indicated by the kinetics and pattern of gene regulation. We examined two key marker genes. Oct4 is a critical determinant of the pluripotent state [ 23] that is downregulated early during somatic differentiation. Fibroblast growth factor (FGF)5 is the earliest characterised marker of ES cell progression to differentiation [ 24]. Oct4 is expressed at similar levels in self-renewing R26NotchIC ES cells as in control ES cells ( Figures 1E and 2J). However, within 24 h of LIF and serum withdrawal, Oct4 mRNA drops by almost two thirds in R26NotchIC cells ( *p* < 0.05, Figure 2J), while there is only a slight decrease in control cells ( Figure 2J). FGF-5 is first expressed in post-implantation epiblast in vivo [ 25]. In vitro, FGF5 mRNA is upregulated transiently at initial stages of lineage commitment of ES cells and then downregulated in definitive germ layer precursors [ 25, 26]. The low level of FGF5 transcript in self-renewing R26NotchIC cells ( Figure 2K [ES]) indicates that they remain in the naïve ES cell state and have not advanced to a more mature egg cylinder stage [ 24]. Control ES cells acquire FGF5 mRNA progressively over several days of monolayer differentiation. In contrast, NotchIC-overexpressing ES cells show a sharp increase in FGF5 mRNA just 24 h after withdrawal of self-renewal factors, consistent with an early and synchronised entry into differentiation ( Figure 2K).

Notch signalling could also act after specification to increase the survival and/or proliferation of neural precursors. We consider that this would be unlikely to contribute significantly to the observed initial increase in frequency of *Sox1*GFP expressing cells, however, because their numbers rise after only 24 h, before the detection of neural specification in control ES cell populations ( Figure 2B). Moreover, the frequency of apoptotic cell death detected by terminal deoxyribonucleotidyl transferase-mediated dUTP-digoxigenin nick end labelling (TUNEL) staining is unchanged at this time and the growth rate of NotchIC and control populations is indistinguishable during the course of the experiment ( Figure S1).

To test further whether Notch directly promotes ES cell differentiation, we examined the density dependence of neural specification in R26NotchIC cultures. Neural induction of ES cells during monolayer differentiation is strongly inhibited by even modest increases in cell density [ 21]. By contrast, R26NotchIC cells generate large numbers of *Sox1*GFP+ neural precursors at densities that almost completely eliminate neural specification in control cultures ( Figure 2L and 2M).

Collectively, these data indicate that NotchIC acts at the initial stages of ES cell differentiation to promote the transition into mature epiblast and subsequently neuroectoderm.

### The Effect of NotchIC Is Not Restricted to 46C ES Cells

ES cells are mutable in culture, and it is therefore important to validate any observed phenotypic effect on more than one cell line. To test whether the effects of NotchIC are generalisable, we took advantage of an independent E14Tg2a derivative, RC, containing the coding sequence for the Cre-ER ^T2^ fusion [ 27] targeted into the *ROSA*26 locus (L. Grotewold and AGS, unpublished data). We targeted our NotchIC expression construct into the second *ROSA*26 locus of RC cells and induced deletion of the transcriptional stop sequence by treatment for 4 d with 1 μM 4-hydroxy-tamoxifen. We then used FACS to collect the NotchIC-CD2+ population and tested these cells in our monolayer differentiation protocol. As with the 46C-derived cell line, RC-derived NotchIC cells began to generate significant numbers of nestin-positive cells with a neuroepithelial morphology during the second day of monolayer differentiation, while almost all control cells remained nestin-negative with an undifferentiated ES cell morphology at this time (unpublished data).

### Requirement for Notch Signalling for Efficient Neural Specification of ES Cells

We next asked whether endogenous Notch signalling regulates neural specification of ES cells during monolayer differentiation. We used both pharmacological and genetic approaches to interfere with Notch activity.

**Figure 3:**
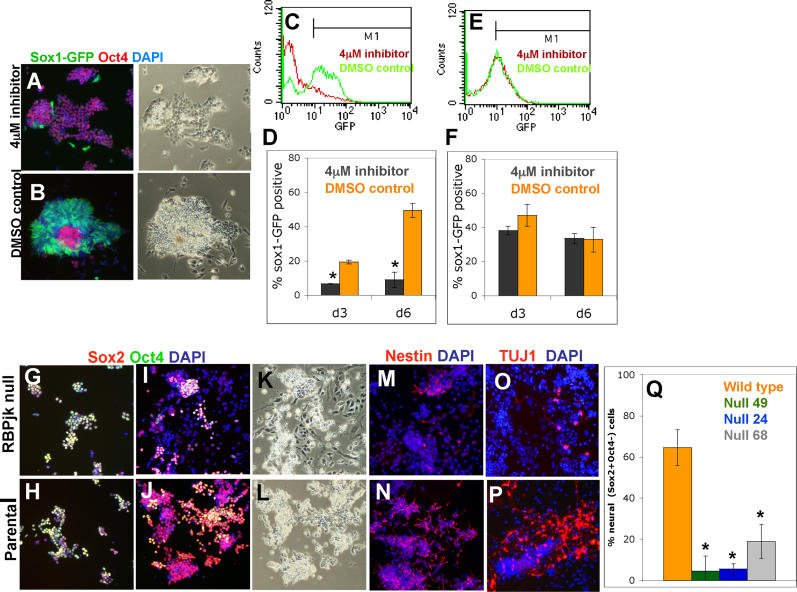
Notch Signalling Is Required for Efficient Neural Specification of ES Cells (A–F) Monolayer differentiation of 46C cells (A–D) or R26NotchIC cells (E and F) exposed to 4 μM γ-secretase inhibitor or to equivalent amounts of DMSO diluent. (A and B) *Sox1*GFP and immunostaining for Oct4 on day 5. (C and E) Typical FACS profiles for *Sox1*GFP on day 6. (D and F) Proportions of *Sox1*GFP+ cells (average of triplicates). (G–P) Monolayer differentiation of RBPJk-null ES cells and the parental D3 ES cell line. Cultures were fixed and stained as indicated on day 2 (G and H) or day 6 (I–P). (Q) Proportion of Sox2+ Oct4− cells on day 6 (average of duplicates).

A critical step in activating Notch is cleavage by γ-secretase to release the intracellular domain. The γ-secretase inhibitor L-685,458 is an effective inhibitor of Notch activation [ 28]. When 46C ES cells are exposed to this γ-secretase inhibitor during the monolayer differentiation protocol, the majority of cells fail to become *Sox1*GFP+ neural cells. Over 6 d of differentiation ( Figure 3), fewer than 10% of inhibitor-treated cells become *Sox1*GFP+ compared with around 50% of cells exposed to DMSO vehicle ( Figure 3A– 3D, *p* < 0.001). Most inhibitor-treated cells remain Oct4 positive during this period ( Figure 3A). Their uncommitted status is confirmed on withdrawal of the inhibitor on day 5, which results in emergence of *Sox1*GFP in the majority of cells over the following 5 d (unpublished data). However, if the inhibitor was maintained beyond 5 d, cells progressively began to differentiate into a nonneural flattened morphology, lacking the neural markers *Sox1*GFP, brain lipid binding protein (BLBP), and nestin such that by the tenth day very few undifferentiated ES cells remain (unpublished data). Our observations suggest that blockade of γ-secretase initially delays lineage commitment and then diverts ES cells into a nonneural fate.

γ-Secretase inhibitors are not specific to Notch, however. These compounds also inhibit membrane cleavage of other molecules, including N-cadherin, ErbB4, and CD44 [ 29]. Therefore, we examined the effect of the inhibitor on R26NotchIC cells. As these cells contain a pre-cleaved NotchIC fragment, they are immune to blockade of Notch cleavage whilst remaining vulnerable to any side effects of the inhibitor. Acquisition of *Sox1*GFP expression by R26NotchIC cells was unaffected by L-685,458 ( Figure 3E and 3F). This establishes that the anti-neural effects of the inhibitor are specifically due to a block in Notch signalling.

Similar experiments were carried out using two alternative neural differentiation protocols: coculture with PA6 stromal cells [ 22] and formation of EBs in the presence of retinoic acid (RA) [ 30]. In both cases, the γ-secretase inhibitor significantly reduced the proportion of cells that became *Sox1*GFP+ ( Figure S2, *p* < 0.05, and unpublished data).

As an alternative loss-of-function approach, we used ES cells that lack the critical downstream mediator of Notch signalling, RBPJk [ 31]. These cells have previously been reported to have some limited capacity for neural differentiation [ 32]. We examined three independent clones of *RBPJk*-null ES cells (No. 24, 49, and 68) plus their parental wild-type line (D3). Since these cells do not contain a *Sox1*GFP reporter and *Sox1* cannot be unambiguously detected by immunohistochemistry, we instead used differential *Sox2* and Oct4 immunostaining to identify neural cells. *Sox2* is a pan-neuroepithelial marker like *Sox1* [ 33], but it also marks undifferentiated ES cells. We carried out double staining for Sox2 and Oct4 and scored cells that were Sox2+ and Oct4− as committed neural precursors. During expansion in LIF plus serum, *RBPJk*-null cells resembled their parental ES cells and uniformly expressed both Oct4 and Sox2. After transferring into N2B27 medium to initiate monolayer neural differentiation, parental cells began to generate Sox2+, Oct4− cells from the third day, peaking at around 60% by day 5 ( Figure 3J, 3L, and 3Q), comparable to differentiation of 46C ES cells ( Figure 2). In contrast, three independent lines of *RBPJk*-null cells generated far fewer (less than 10%) Sox2+, Oct4− cells ( Figure 3I, 3K, and 3Q, *p* < 0.05). Cell death was minimal in both *RBPJk*-null and control populations. Similar to the cells treated with the pharmacological Notch inhibitor, the *RBPJk*-null ES cells either retained Oct4, indicating failure to differentiate, or lost expression of both markers and became larger and flatter, indicating nonneural differentiation. The proportions of nestin+ cells, Pax6+ cells, and BLBP+ cells were also much lower in the *RBPJk-*null cells compared with control cultures ( Figures 3M, 3N, and S3). Furthermore, we noted that many of the RBPJk nestin+ cells had a large flat morphology and were negative for Sox2, indicating that they are not neural progenitors. This is consistent with previous evidence that nestin is not an exclusive neural marker [ 34, 35] and should be used with caution in attributing neural identity. Finally, in contrast to the plentiful TuJ1+ cells generated by parental cells after 5 d of monolayer differentiation, *RBPJk*-null cells generated only a few small foci of TuJ1+ cells in keeping with the dramatic reduction in the number of neural progenitors ( Figures 3O, 3P, and S3).

Therefore, both pharmacological inhibition and genetic disruption of Notch signalling severely reduce commitment of ES cells to the neural lineage, although neither completely blocks neural fate (see Discussion).

### Notch Acts in Combination with FGF Signalling to Promote Neural Specification

**Figure 4:**
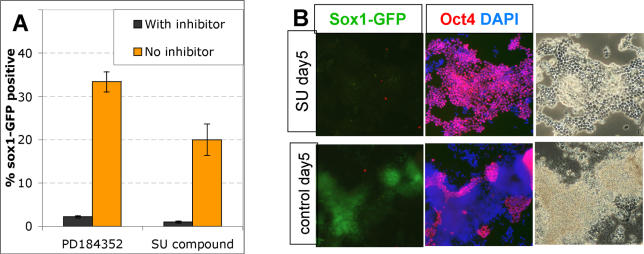
NotchIC Does Not Bypass the Requirement for FGF Signalling in Neural Specification (A) R26NotchIC cells or parental control 46C cells were cultured in triplicate for 3 d in the presence of 4 μM PD184352, 5 μM SU5402, or in an equivalent concentration of DMSO diluent (“No inhibitor”) and the proportion of *Sox1*GFP+ cells analysed by FACS on day 3. (B) Intact cultures were immunostained for Oct4 on day 5 to visualise undifferentiated ES cells.

In vertebrate embryos, neural induction requires FGF signalling through the Ras-Erk pathway [ 36– 38]. This also appears to be true for ES cells, where the initial stimulus is most likely provided by autocrine FGF4 [ 21]. We tested whether NotchIC could bypass this requirement using two pharmacological inhibitors ( Figure 4). SU5402 inhibits the FGF receptor tyrosine kinase [ 39], and PD184352 blocks Mek1/2, upstream of Erk1 and Erk2 [ 40]. These inhibitors are both able to block neural specification of ES cells [ 7, 8] (unpublished data). R26NotchIC cells remain susceptible to blockade of neural induction by these inhibitors. In the presence of these agents they continue to proliferate in an apparently undifferentiated Oct4+ state for at least 5 d with minimal generation of *Sox1*GFP+ derivatives ( Figure 4B). These findings indicate that FGF and Notch act combinatorially in the neural induction cascade. It remains to be determined whether Notch is activated independently of or consequent to FGF signalling.

### NotchIC Cannot Overcome the Serum Inhibition of Neural Differentiation

Serum is a potent antagonist of neural differentiation, but addition of 5 μM RA to differentiating embryoid bodies (EBs) overcomes this inhibitory effect by unknown mechanisms [ 30]. Interestingly, although inhibition by the γ-secretase inhibitor suggests that Notch is important for RA-induced neural differentiation, we found that NotchIC is not sufficient to substitute for RA for neural differentiation of EBs in serum ( Figure S4A). We noticed, however, that RA-treated R26NotchIC EBs contained uniformly distributed *Sox1*GFP+ cells, in contrast with control EBs, which contained patches of *Sox1*GFP+ cells and were surrounded by a *Sox1*GFP− outer layer ( Figure S4). Thus, NotchIC appears to have a similar effect in homogenising the distribution of *Sox1*GFP+ cells generated during two different types of differentiation protocols: EB culture in the presence of RA and serum, and serum-free monolayer culture (compare Figure S4A with Figure 2I). This may indicate that part of the mechanism by which RA acts could be to liberate the Notch pathway from the suppressive effects of serum.

### R26NotchIC Neural Precursors Transit from *Sox1*+ to *Sox1*− Status

**Figure 5:**
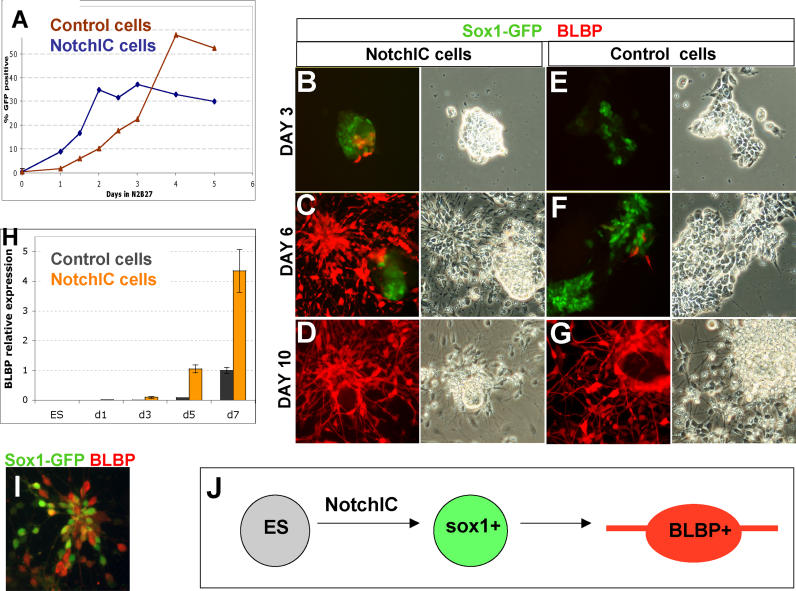
R26NotchIC Cells Lose *Sox1* and Acquire BLBP Expression R26NotchIC ES cells or parental control 46C cells cultured under monolayer differentiation conditions. (A) Proportion of *Sox1*GFP+ cells at various time points measured by FACS analysis. (B–G) Cultures at various time points shown in phase contrast or stained for markers as indicated. (H) Quantitative RT-PCR for BLBP normalised to GAPDH and displayed relative to expression in day 7 46C ES cells. (I) R26NotchIC cells at day 7 of monolayer differentiation stained with an antibody to GFP in order to amplify detection of *Sox1*GFP (green) together with an antibody to BLBP (red). (J) Schematic diagram to illustrate the transition of ES cells into *Sox1*GFP+ neuroepithelial progenitors and then into BLBP+ bipolar neural progenitors.

During parental 46C-cell lineage commitment ( Figure 5), *Sox1*GFP+ cells do not reach a maximum until the fifth day and then remain at this plateau for several days ( Figure 5A). In contrast, the proportion of *Sox1*GFP+ cells in R26NotchIC cultures peaks by day 3 and begins to decline soon afterwards ( Figure 5A). On day 3, R26NotchIC cells first begin to express BLBP, a marker of more mature neural precursors that appears much later in parental cell differentiation ( Figure 5B). This is followed between day 4 and day 6 by transition to bipolar morphology in many of the cells ( Figure 5C). These bipolar cells are immunopositive for BLBP ( Figure 5C and 5D) and RC2 (not shown) and negative for GFAP, characteristics of neural precursor cells in vivo and of neural stem (NS) cells in vitro [ 41, 42]. By the fifth day, at least 50% of NotchIC cells express BLBP, compared with fewer than 5% of control cells. Quantitative RT-PCR confirmed that BLBP was induced in R26NotchIC cells much earlier than in control cells ( Figure 5H). Control ES cells similarly only develop bipolar morphology at later time points ( Figure 5F and 5G). *Sox1*GFP expression is downregulated as BLBP is upregulated, such that the two markers are generally mutually exclusive. However, we do observe some BLBP+ cells at early time points in R26NotchIC differentiation that coexpress weak levels of GFP, likely due to perdurance of GFP protein ( Figure 5I). These observations suggest that R26NotchIC-derived early neural cells rapidly progress through the *Sox1*+ pan-neuroepithelial stage and convert to *Sox1*− BLBP+ neural cells. This explains why numbers of *Sox1*GFP+ cells do not continue to increase beyond the third day in monolayer differentiation.

### NotchIC Suppresses Nonneural Differentiation

**Figure 6:**
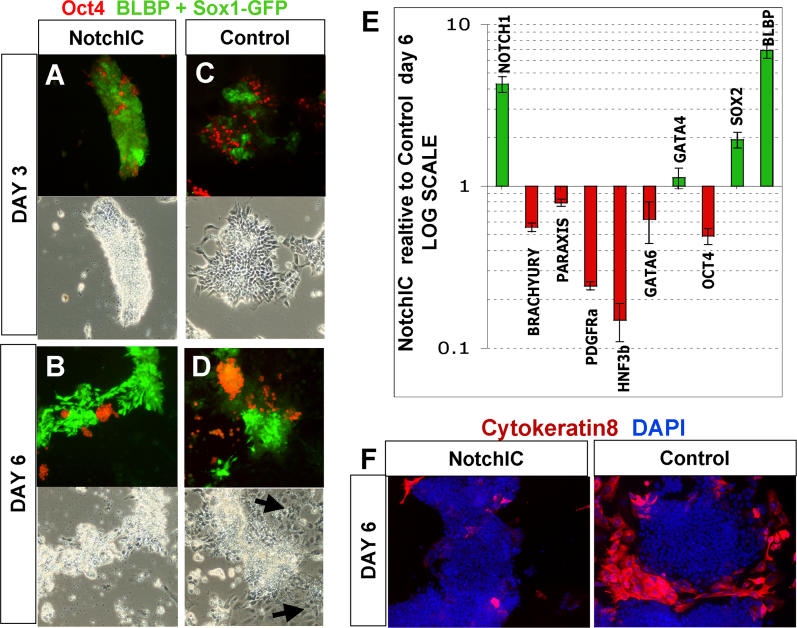
NotchIC Inhibits Nonneural Differentiation (A–D) R26NotchIC cells (NotchIC) or parental 46C cells (control) cultured under monolayer differentiation conditions and stained for Oct4 (red) to indicate ES cells together with a combination of BLBP and GFP (green) to indicate both types of neural progenitor together. (E) qRT-PCR for various markers in R26NotchIC cells relative to parental 46C cells after 6 d of monolayer differentiation. Data are averaged from at least three independent experiments and normalised to GAPDH. Notch1 primers amplify within the NotchIC and detect both endogenous and exogenous transcripts. (F) R26NotchIC cells (NotchIC) or parental 46C cells (control) cultured under monolayer differentiation conditions and stained for cytokeratin 8, a marker of nonneural differentiation (red) and counterstained with DAPI (blue)

In Figure 6A– 6D, we used a combination of BLBP immunostaining and *Sox1*GFP, both visualised together in green in order to show all neural progenitors, with Oct4 immunostaining in red to indicate undifferentiated ES cells. In NotchIC cultures, the vast majority of the cells express neural markers by day 3 ( Figure 6A). Only a minor subpopulation resists neural differentiation (less than 10%) and persists as undifferentiated ES cells. Practically all cells can be accounted for by expression of either neural or ES markers at either day 3 or day 6 ( Figure 6A and 6B). This contrasts with control cultures, where there are more persisting ES cells, but in addition, 15% to 30% of cells lack neural or ES cell markers and exhibit nonneural differentiated morphologies by day 6 ( Figure 6D). Many of these cells express cytokeratin 8, a differentiation marker that is not present in ES cells or neural lineages ( Figure 6F). We carried out quantitative RT-PCR to measure the expression of endoderm and mesoderm markers on the sixth day of monolayer differentiation ( Figure 6E). Several nonneural markers were readily detected in parental cell samples, in marked contrast to the barely detectable expression levels in R26NotchIC cell. These observations indicate that not only does Notch promote neural lineage entry but it also simultaneously suppresses nonneural commitment.

R26-NotchIC ES also showed a marked reduction in mesoderm differentiation when tested under an inductive differentiation protocol based on monolayer culture on collagen IV in the presence of batch-tested serum [ 4] ( Figure S5).

### NotchIC Allows for the Maintenance of BLBP+ Cells Rather than Promoting Their Rapid Terminal Differentiation into Astrocytes or Neurons

The Notch signalling pathway has been reported to either promote expansion of neural progenitor cells [ 43], or to promote their differentiation into astrocytes [ 44], depending on the developmental stage and context. We observed that the majority of NotchIC cells persist as BLBP+ nestin+ RC2+ cells for at least 3 wk in the absence of added growth factors ( Figure S6E, S6G, and unpublished data). During the second week of culture, neuron-like cells that stained with TuJ1 appeared ( Figure S6A) but in fewer numbers than in control cultures and significantly outnumbered by undifferentiated BLBP+ cells ( Figure S6B, S6H, and S6I). GFAP immunopositivity, indicative of astrocyte differentiation, was rarely observed until the third week and even then only in a low number of cells ( Figure S6K). However, upon transfer to serum-containing medium, the R26NotchIC BLBP+ cells efficiently differentiate into GFAP+ astrocytes ( Figure S6C). We conclude that R26NotchIC restrains but does not irreversibly block terminal differentiation.

### Notch Promotes Neural Specification in Human ES Cells

Notch pathway genes have been reported to be expressed in human ES cells [ 45, 46]. We therefore investigated whether the role of Notch in neural differentiation is conserved in human ES (hES) cells. We first confirmed that the Notch ligands Jagged1, Jagged2, Delta1, and Delta3 could all be readily detected in human ES cells by RT-PCR ( Figure S7). Mis-expression of Notch ligands in feeder cells has been shown to activate Notch in other cell types [ 47, 48], so we decided to employ this strategy with hES cells.

**Figure 7:**
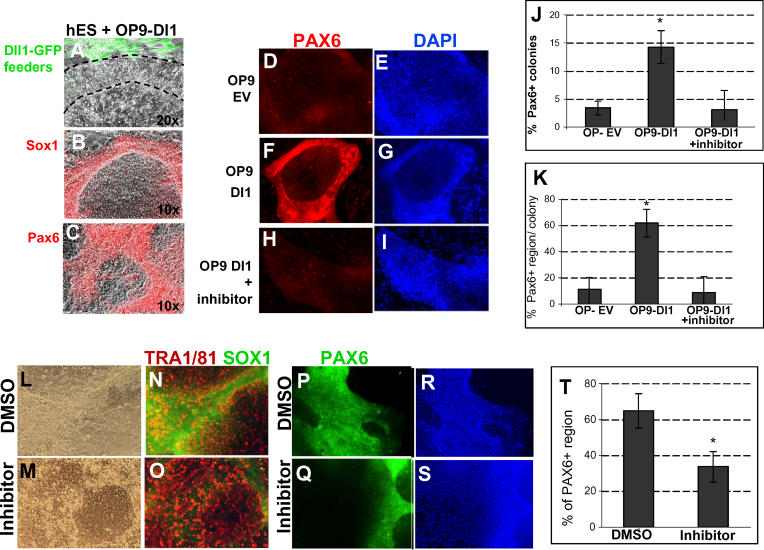
Notch Promotes Neural Differentiation of Human ES Cells (A–I) Human ES cells plated on OP9 feeder cells expressing either GFP only (OP9 EV) or the Notch ligand Delta1 (OP9 Dl1) with γ-secretase inhibitor where indicated (“+inhibitor”) were cultured for 7 d and stained for markers as indicated. (A) Higher magnification picture to indicate the cell morphology: the OP9-Dl1 feeders are green and the dotted line demarcates hES cells adjacent to the feeders that have adopted a neural morphology, while cells more distant from the feeders retain an ES-like morphology. (J and K) Quantification of Pax6 immunostaining (averages and standard deviations shown from four experiments). (L–T) Human ES cells grown under monolayer differentiation conditions in the presence of γ-secretase inhibitor (“inhibitor”) or DMSO vehicle and stained for Pax6, *Sox1*, or TRA1/81 as indicated. T shows quantification of Pax6 immunostaining (averages and standard deviations from four experiments).

We made use of OP9 cells that stably express the Notch ligand Delta1 together with GFP, through retroviral transduction (OP9-Delta1) [ 49]. Control OP9 feeder cells had been transduced with a GFP-only retrovirus (OP9-EV). When hES cells were plated onto OP9-EV feeder layers in serum-free medium containing bFGF but no LIF or BMP4, the majority of cells maintained an ES-like morphology after 1 wk. In contrast, when cells were plated onto OP9-Dl1 feeders in the same medium, the edges of the colonies, where they contact the GFP+ OP9-Dl1 feeder cells, underwent a morphological change within the first week ( Figure 7). They became compact and elongated with barely distinguishable nuclei ( Figure 7A, region between dotted lines). Antibody staining confirmed that these cells were negative for the ES markers TRA1/60 and TRA 1/81 (unpublished data) and positive for *Sox1,* Nestin, and Pax6 ( Figure 7B, 7C, and unpublished data). In human ES cells, Pax6 is the earliest known marker of neural differentiation, appearing several days before *Sox1* begins to be expressed [ 50, 51]. We carried out quantification of this marker using image analysis software. This confirmed a significant increase in both the number of Pax6+ colonies (colonies containing more than ten Pax6+ cells) and in the area that is Pax6+ within each of these colonies in OP9-Dl1–supported cultures in comparison with OP9-EV colonies ( Figure 7D– 7G, 7J, and 7K, *p* < 0.01). The positive effect of OP9-Dl1 feeders on neural differentiation appeared to be specifically due to activation of Notch signalling, because it could be blocked by adding the gamma secretase inhibitor ( Figure 7H– 7K, *p* < 0.01). Exposure to neither Delta1 nor the γ-secretase inhibitor had any discernible effect on cell number or viability.

We went on to test whether endogenous Notch signalling is required for neural differentiation in hES cells. For these experiments, we made use of a monolayer neural differentiation protocol adapted from that for mouse ES cells. Briefly, we removed the hES from feeders and from exogenous LIF and BMP4 and plated them onto Matrigel in FGF–only serum-free medium. Under these conditions, typically around 60% of the culture area loses expression of the ES cell marker TRA1/81, adopts a neural morphology, and becomes *Sox1*+ Pax6+ after 1 wk ( Figure 7L, 7M, 7P, 7R, and 7T). In contrast, in the presence of the γ-secretase inhibitor, there is a significant reduction in emergence of Pax6+ regions within the culture ( Figure 7M, 7O, 7Q, 7S, and 7T, *p* < 0.5), with a corresponding increased persistence of undifferentiated TRA1/81+ ES cells ( Figure 7O). The γ-secretase inhibitor had no obvious effect on cell viability or cell number, and the majority of treated cells retained a healthy hES-like morphology ( Figure 7M).

These data suggest that Notch plays a similar role to promote neural differentiation in human ES cells as it does in mouse ES cells.

## Discussion

Pluripotent ES cells should be a valuable source of neural cell types for cell biological investigation, neurodegenerative disease modelling, pharmaceutical screening, and possibly even regenerative therapies. If ES cells are to be harnessed effectively for these goals, it will be necessary to develop robust methods for directing neural commitment and suppressing differentiation into other lineages. In this study we have presented evidence for an unsuspected role of the Notch signalling pathway in promoting and directing primary fate choice in ES cell differentiation. Activation of Notch thus emerges as a key tool for steering ES cells toward the neural fate and away from nonneural fates. Although we have relied primarily on genetic manipulations to activate Notch, it should in principle be possible to achieve the same result by adding specially engineered soluble forms of Notch ligands or other forms of Notch agonist to the culture medium [ 52].

In the presence of LIF, moderate expression of an activated form of Notch does not interfere with the self-renewal of ES cells. However, after removal of self-renewal signals, NotchIC significantly increases the rate and frequency of neural specification in serum-free media whilst blocking differentiation into nonneural lineages. Conversely, pharmacological inhibition of Notch signalling or genetic ablation of RBPJk significantly reduce the proportion of ES cells that enter the neural lineage, temporarily arresting differentiation and then diverting ES cells into nonneural fates. These data suggest that endogenous Notch signalling both mediates and is a limiting factor for neural specification in ES cell cultures. In addition, we find that moderate levels of activated Notch from the R26 locus promotes the generation and expansion of BLBP+ neural cells and suppresses terminal neuronal differentiation without promoting astrocyte differentiation. Thus, the Notch pathway directs lineage commitment in ES cells but restrains differentiation in neural stem/progenitor cells.

Previously we have reported that autocrine FGF signalling promotes neural conversion of ES cells in the absence of serum, BMP, or Wnts [ 7, 17, 53]. The FGF signal does not appear to be limiting for this lineage choice since added FGF has little effect except at low cell densities [ 21]. R26NotchIC in contrast enhances neural specification over a range of cell densities. However, it is not dominant over all other signals. Notch is not sufficient to drive neural specification in the presence of serum and in the serum-free monolayer protocol the effect of R26NotchIC is diminished at very high cell densities. Furthermore, even at standard cell densities, 5% to 10% of cells still resist differentiation. This is approximately half the number observed for control cultures but nonetheless raises the question why do some ES cells elude neural commitment? These cells are not differentiation defective as they generally undergo neural specification if replated into a fresh monolayer differentiation. Possibly Notch responsiveness oscillates in ES cell cultures as reported for embryonic tissues [ 54] or occasional predifferentiated cells in the cultures secrete sufficiently high local levels of LIF and/or BMP to sustain local self-renewal [ 55].

Forced expression of *Sox1* in ES cells has a similar effect to NotchIC in promoting more uniform neural specification [ 56]. The pharmacological Notch inhibitor appears to have little or no impact on the initial onset of neural specification in the context of *Sox1* overexpression (unpublished data). This suggests that *Sox1* is downstream of Notch in the neural induction cascade and further supports the contention that Notch acts in the primary phase of neural specification in undifferentiated *Sox1*− ES cells.

During neural differentiation a proportion of *Sox1*+ neuroepithelial cells mature into bipolar *Sox1*−, BLBP+, GFAP− neural precursor cells with certain features of radial glia [ 42]. NotchIC appears to promote this transition, with BLBP+ cells appearing earlier and in high numbers. In vivo studies indicate that an activated form of Notch increases the number of radial glia that emerge from the neuroepithelium in vivo [ 57] and from neuroepithelial cells in culture [ 58] and that BLBP is a direct target of Notch signalling in radial glial cells [ 59]. It is also possible that the precocious generation of a BLBP+, GFAP− phenotype in the presence of NotchIC is purely secondary to the accelerated conversion of ES cells into neuroectoderm. However, the lower peak proportion of *Sox1*GFP+ cells suggests that they do not accumulate in this phase to the same extent as parental cells. Thus, Notch appears to drive differentiation forward at both the ES cell and neuroectodermal precursor stage, but then to restrain terminal differentiation.

BLBP+ cells act as neuronal precursor cells in vivo [ 59] and in culture [ 42]. This raises the question of whether the bulk of our R26-NotchIC ES-derived cells retain neuronal as well as astrocyte differentiation potential. We do observe that a minor subpopulation of these cells spontaneously differentiate into neurons during the course of our experiments ( Figure S6). However, NotchIC is well established as an inhibitor of overt neuronal differentiation [ 9], and so it is not possible to assess the true neuronal differentiation capacity whilst R26-NotchIC is overexpressed. Future studies using a cre-revertable or tet-regulatable NotchIC expression system should make it possible to directly address whether sustained exposure to active Notch restricts ES-derived neural cells to an astrocyte fate or maintains neurogenic competence.

Significantly, we find no acceleration of astrocyte differentiation in response to NotchIC in our experiments. Notch has been reported to promote astrocyte terminal differentiation, but this appears to be an age-dependent phenomenon. For example, NotchIC drives astrocyte differentiation in adult hippocampal cells [ 44] but not in earlier neural progenitors, for example from E11.5 embryonic cortex [ 60].

Notch has been shown to act in other contexts to promote neural competence in tissues that would otherwise become nonneural. Subsequently in the same tissue, Notch can inhibit final differentiation. In the *Drosophila* eye, Notch has such sequentially distinct effects, first promoting neural competence and later inhibiting overt neural differentiation [ 61]. Similarly, in the developing inner ear of the chick activated Notch promotes generation of sensory patches followed by a later function to inhibit the differentiation of sensory hair cells within those sensory patches [ 11]. Interestingly, Jagged1 appears to be the Notch ligand responsible for mediating this pro-sensory role of Notch [ 11]. We find that Jagged1 is expressed in undifferentiated ES cells, consistent with this being the Notch ligand that drives neural specification. Furthermore, the fact that Jagged1 is expressed only in around half of cells may explain why Notch activity remains limiting.

In the developing embryo, neighbouring cells must continually communicate with each other to coordinate patterning of tissues. Notch signalling patterns tissues by at least two types of mechanism. Lateral inhibition mechanisms ensure that neighbouring cells follow different fates, so that one single cell type does not dominate within a particular region [ 10]. Lateral induction, a form of community effect [ 54], acts in the opposite way to ensure that cells within a particular region adopt the same fate choice. In the context of neural induction from ES cells, our findings indicate that Notch signalling acts to amplify and consolidate neural specification.

The key features of Notch in ES cell fate choice are that it (1) synchronises the timing of neural specification, (2) homogenises the levels of *Sox1* between different cells within the population, and (3) inhibits nonneural differentiation. These observations provoke consideration of a possible role for Notch signalling in vertebrate germ layer specification. Neural induction in vivo is regulated by an interplay between FGF, BMP, and Wnt signals [ 62], but recent data indicate that a further, as yet unidentified signalling event is also required [ 63]. Our ES cell data raise the possibility that Notch may be a component of this circuit.

In the mouse embryo, Notch pathways mutants have not been reported to show defects in gastrulation or primary neural specification [ 64]. Requirements for Notch signalling may be masked by redundancy, especially in the case of the Notch family itself, which has four members. Redundancy is, however, less likely to be an issue for RBPJk, which is thought to mediate the downstream response to all four Notches (although Notch can signal via RBPJk-independent pathways in some contexts [ 65]). *RBPJk-*null embryos develop multiple abnormalities around E8, dying soon afterward [ 66]. A nestin-positive neuroepithelium is present in these embryos but is reported to be thinner. The mutants are also characterised by failure of anterior neural tube closure. Therefore, examination of earlier stages in these embryos could reveal deficiencies in generation of neural tissue. Our analysis of the RBPJk mutant ES cells indicates that they are severely deficient, although not entirely blocked, in their ability to generate neural cells. These data are supported by experiments with the γ-secretase inhibitor. Furthermore, *Notch1*-null or *RBPJk*-null ES cells appear biased towards mesodermal differentiation in both embryoid body and stromal coculture systems (F. Ratke and T. Schroeder, personal communication).

Nonetheless, RBPJk-dependent Notch signalling appears to be more significant for neural specification in ES cells than for neural induction in vivo. The ES cell data suggest a role of Notch not as a primary inducer but as an amplifier that coordinates uniform neural induction within a population, helping to both synchronise the timing with which cells respond to inductive cues, notably FGF, and to protect against nonneural differentiation in face of fluctuations in self-renewal and differentiation signals. This is reminiscent of the role of Notch during somitogenesis, coordinating the timing of cyclic gene expression within each somite [ 67]. Such a coordination role may be of greater significance for cultured ES cells, confronted with conflicting autocrine, paracrine, and exogenously provided differentiation cues, than in the gastrulating embryo where differentiation signals are tightly restricted, topologically and temporally. Indeed, the challenge in ES cell biology is precisely to suppress the diversity of embryonic development and impose unitary lineage commitment. We suggest that manipulation of the Notch pathway may be a key component in achieving this goal.

## Materials and Methods

All experiments were carried out at least three times with similar results, unless otherwise stated. Error bars represent standard deviations, and *p* values are calculated using a two-tailed Student's *t*-test.

### Targeting NotchIC into 46C ES cells

Our targeting construct is based on similar constructs previously used for targeting into the *ROSA* locus [ 16]. It contains a *pgk-neo* cassette plus a triple-polyA termination sequence, which together are flanked by *loxP* sites. This is followed by the coding sequence for the intracellular domain of NotchIC [ 14] followed by an internal ribosomal entry site followed by the coding sequence for the human cell surface molecule CD2. This construct was transfected into 46C ES cells [ 7] or into R26-Cre-ER ^T2^ (RC) cells (L. Grotewald and AGS) by electroporation, and clones were expanded under G418 selection. Correctly, targeted clones were identified by Southern blotting after digestion with EcoRV. The targeted allele gives an 11-kb band, while the wild-type allele gives a 3.8-kb fragment. Clones of targeted 46C cells were transfected with a plasmid containing CRE in the CAGS expression unit [ 68]. Clones of targeted R26-Cre-ER ^T2^ cells were exposed to 1 μM 4-hydroxy-tamoxifen for 4 d to induce CRE activity. Cells that failed to undergo CRE-mediated deletion of the pgk-neo STOP cassette were eliminated by FACS collection of the CD2-positive population.

### Luciferase assay

The 12xRBPJk-luciferase Notch reporter construct (gift from U. Lendahl and E. Hansson) was originally described in [ 69]. Luciferase assays were carried out using a Dual Luciferase Reporter Assay System (Promega, Madison, Wisconsin, United States).

### Mouse ES cell culture

ES cells were maintained in Glasgow modification of Eagle's minimal essential medium supplemented with 2-mecaptoethanol, nonessential amino acids, sodium bicarbonate, 10% FCS, and 100 units/ml LIF on gelatinised tissue culture flasks [ 70].

### Mouse ES cell monolayer differentiation

This was described in detail in [ 7]. Briefly, ES cells were washed to remove all traces of serum and then plated onto gelatin-coated tissue culture plastic at a density of 1 × 10 ^4^ cells/cm ^2^ in N2B27 serum-free medium. N2B27 consists of a 1:1 ratio of DMEM/F12 and Neurobasal media supplemented with 0.5% modified N2 (made in house as described in [ 7], 0.5% B27 (GIBCO, San Diego, California, United States), and 2-mercaptoethanol. Medium was changed every second day. For some experiments, cells were replated after 7 d onto dishes coated with laminin and polyornithine. For astrocyte differentiation, the culture medium was supplemented with 10% FCS after 14 d of serum-free culture.

### Other differentiation protocols

Coculture with PA6 stromal cells was carried out essentially as described previously [ 22] except that the serum-free medium used was N2B27 as described above. Generation of EBs was carried out as previously described [ 30]. RA was added on the fourth day of EB culture. Mesoderm differentiation was carried out as previously described [ 4].

### Pharmacological inhibitors

The ERK inhibitor PD184352 [ 40] (kind gift of P. Cohen, University of Dundee) was used at a concentration of 4 μM. The FGF receptor tyrosine kinase inhibitor SU5402 [ 39] was used at 5 μM. The γ-secretase inhibitor (cat. 565771; Calbiochem, San Diego, California, United States) was used at a concentration of 4 μM. None of these inhibitors had any obvious toxic effects over the time course of the experiments.

### Human ES cell culture and differentiation

Undifferentiated human ES cells (line 181 p99-p130 [ 71]) were maintained on a layer of human foreskin fibroblast (line HS27; ATCC, Manassas, Virginia, United States) in the defined medium N2B27 supplemented with LIF (10 ng/ml), BMP-4 (3 ng/ml; R & D Systems, Minneapolis, Minnesota, United States), and bFGF (10 ng/ml; R & D Systems). Cells were passaged at a split ratio of 1:2 every week using collagenase IV (1 mg/ml). Feeder-free neural differentiation was performed following the monolayer protocol used for mouse cells, modified to suit human ES cells as follows: cells reaching 50% confluence were incubated in collagenase IV for 15 min, washed once in PBS and detached in N2B27 medium supplemented with bFGF (FGF medium, no LIF, no BMP4) using glass beads. Cells were then incubated for 4 h in a gelatinised flask in the same medium to allow the differential attachment of the feeder cells. Finally, the ES suspension was plated at a 1:1 ratio in culture dishes pre-coated with Matrigel (low growth factor Matrigel, 1:20; BD Biosciences PharMingen, San Diego, California, United States). When indicated, the γ-secretase inhibitor (4 μM) was added from the start of the feeder-removal step and then added every other day when the medium was changed. For coculture with OP-9 cells, hES cells were treated with collagenase as described above and then manually detached to avoid carry-over of human fibroblasts. Cells were then directly plated in FGF medium on a layer of γ-irradiated OP9-EV or OP9-Dl1 stromal cells (kindly provided by A. Cumano). Matrigel was used to promote the survival of the OP9 in the serum-free medium. The γ-secretase inhibitor was added at plating and then added every other day when the medium was changed.

### Quantification of neural differentiation of human ES cells

Cells were processed for immunocytochemistry and neural differentiation quantified as follows. For OP9 coculture experiments, the number of colonies with positive PAX-6 cells was counted and normalised to the total number of colonies in the well. For all experiments (feeder-free and OP9-dependent differentiation), Volocity image analysis software (Improvision, Lexington, Massachusetts, United States) was used to quantify the extent of differentiation. Briefly, the software was used to calculate the area of the well (feeder-free experiments) or of each colony (OP9 experiments) covered by PAX-6–positive nuclei. The values were then normalised to the area covered by the cells or colonies, using DAPI staining. All experiments were repeated at least three times with four wells per condition.

RT-PCR primers used for RT-PCR are described in Table S1.

### Immunofluorescence and FACS

Cells were fixed in 4% paraformaldehyde and incubated for 30 min in blocking buffer (PBS, 3% goat serum, and 0.1% Triton X-100). Primary antibodies were diluted in blocking buffer and applied for 1 h at room temperature or overnight at 4 °C. After three washes in PBS, secondary antibodies conjugated to Alexa fluorophores (Molecular Probes, Eugene, Oregon, United States) were diluted at 1:1,000 in blocking buffer and applied for 1 h at room temperature. The cells were washed at least three times in PBS and visualised on an Olympus inverted fluorescence microscope. For nuclear counter staining, cells were incubated in 10 μg/ml DAPI (Sigma, St. Louis, Missouri, United States) for 10 min after immunostaining.

In experiments where cells were counted, nuclei were counterstained with DAPI, at least 1,000 cells per dish were counted from three separate dishes, and an average was taken. Primary antibodies were obtained from the following sources: human CD2 (BD Biosciences), Oct4 (sc-5279; Santa Cruz Biotechnology, Santa Cruz, California, United States), GFP (Molecular Probes), nestin (Developmental Studies Hybridoma Bank [DSHB]), *Sox2* (Chemicon, Temecula, California, United States), BLBP (gift of N. Heintz); neuronal beta-III tubulin (Covance, Madison, Wisconsin, United States), GFAP (Sigma), TRA 1/60 and TRA 1/81 (Chemicon), human *Sox1* (Chemicon), human Pax6 (Covance), O4 (DSHB), RC2 (DSHB), Nanog (gift of I. Chambers), cytokeratin 8 (DSHB), Flk1 (AVAS12; gift of S. Nishikawa [ 72]), PDGFRα (APA5; gift of S Nishikawa [ 73]), and E-cadherin (ECCD2; gift of M. Takeichi [ 74]).

Single chain antibodies against Jagged1 and Desmin were generated by the Atlas group at the Sanger Institute (J. McCafferty and J. Young) as part of the Framework VI Integrated Project EuroStemCell.

Antibodies obtained from the Developmental Studies Hybridoma Bank were developed under the auspices of the National Institute of Child Health and Human Development and maintained by The University of Iowa, Department of Biological Sciences, Iowa City, Iowa, United States. TUNEL staining was performed using a DeadEnd Fluorometric TUNEL detection kit (Promega).

FACS analysis was performed using a Becton-Dickinson (Palo Alto, California, United States) FACS Calibur flow cytometer. FACS sorting was carried out on a Dako (Glostrup, Denmark) Cytomation MoFlo High Performance Cell Sorter.

## Supporting Information

Figure S1 Cell Death and Proliferation (A) Growth curve indicating the total number of cells at various time points in triplicate monolayer differentiation cultures (average of triplicates). (B) Proportion of TUNEL-positive cells after 24-h monolayer differentiation (average of triplicates).
(203 KB TIF)

Figure S2 γ-Secretase Inhibitors Interfere with Neural Specification during Alternative Neural Differentiation Protocols (A–C) R26NotchIC cells or 46C parental control cells were cultured for 8 d under the PA6 differentiation protocol in the presence of 4 μM γ-secretase inhibitor or the equivalent volume of DMSO. (A, B) *Sox1*GFP expression in live colonies (C) quantification of the proportion of colonies containing GFP-positive cells (average of three independent experiments). (D) R26NotchIC cells or 46C parental control cells were cultured under the embryoid body plus RA protocol for 8 d in the presence of 4 μM γ-secretase inhibitor or the equivalent volume of DMSO, and the proportion of *Sox1*GFP+ cells was quantified by FACS analysis after disaggregation of EBs (average of triplicates).
(1.7 MB TIF)

Figure S3 Neural Markers in RBPJk-Null Cells after 6 d under the Monolayer Differentiation Protocol Monolayer differentiation of RBPJk-null ES cells and the parental D3 ES cell line. Cultures were fixed and stained as indicated on day 6.
(7.9 MB TIF)

Figure S4 NotchIC Does Not Bypass the Requirement for Retinoic Acid in Neural Specification in the Presence of Serum R26NotchIC cells or 46C parental control cells were cultured as EBs (EBs) in the presence or absence of RA. (A) Typical FACS profile for *Sox1*GFP in a population of cells from disaggregated EBs. (B and C) Distribution of *Sox1*GFP in live EBs.
(1.8 MB TIF)

Figure S5 NotchIC Suppresses Mesoderm Lineage Choice FACS analysis of mesoderm markers PDGFRα and Flk1 in NotchIC or parental controls cells after 5-d culture of collagen IV–coated dishes in the presence of serum..
(3.1 MB TIF)

Figure S6 R26NotchIC Cells Generate Neurons and Astrocytes, but These Are Outnumbered by Undifferentiated BLBP+ Cells unless Cells Are Exposed to Serum (A and B) Intact cultures at day 7 of monolayer differentiation, stained for markers as indicated. (C) Cells replated onto gelatin after 7-d monolayer differentiation, cultured for a further 7 d in the absence of serum and then for the final 7 d in the presence of serum and 100 units/ml LIF and then fixed and stained for GFAP. (D–K) Cells replated onto laminin after 7 d of monolayer differentiation and cultured for a further 14 d and then fixed and stained for markers as indicated.
(6.2 MB TIF)

Figure S7 Expression of Notch Ligands in Human ES Cells RT-PCR for Notch ligands in human ES cells. Human foetal brain tissue was used as a positive control.
(628 KB TIF)

Table S1 Primers Used for RT-PCR (25 KB DOC)

## Abbreviations

BLBP: brain lipid binding protein
BMP: bone morphogenetic protein
CRE: Cre recombinase
ES: embryonic stem
FACS: fluorescence activated cell sorter
FGF: fibroblast growth factor
GFP: green fluorescent protein
hES: human embryonic stem
LIF: leukemia inhibitory factor
NotchIC: Notch intracellular domain
NS: neural stem
RA: retinoic acid
RBPJk: recombination binding protein J kappa
TUNEL: terminal deoxyribonucleotidyl transferase-mediated dUTP-digoxigenin nick end labelling
