# Supplementary material for: Notch Promotes Neural Lineage Entry by Pluripotent Embryonic Stem Cells
Source: PLoS Biol. 2006 Apr 11;4(5):e121. doi: 10.1371/journal.pbio.0040121 (PMC1431581; doi:10.1371/journal.pbio.0040121)
Supplement: Supplementary file 1 [file pbio.0040121.st001.doc]

Lowell et al Supplementary Table 1

|  | Forward primer | Reverse primer |
| --- | --- | --- |
| GAPDH | CCCACTAACATCAAATGGGG | CCTTCCACAATGCCAAAGTT |
| Notch1 | CCAGCATGGCCAGCTCTGG | CATCCAGATCTGTGGCCCTGTT |
| Notch2 | CCAGGATTCACAGGAGAGGA | CATTTTCGCAGGGATGAGAT |
| mJagged1 | GTCCACG GCACCTGCAATG | CAAGGTTTGGCCTCGCACT |
| hJagged1 | TCGGGTCAGTTCGAGTTGGA | AGGCACACTTTGAAGTATGTGTC |
| mJagged2 | TGGGTGGCAACTCCTTCTAC | CCTCATCTGGAGTGGTGTCA |
| hJagged2 | AGCTGGACGCCAATGAGTG | GTCGTTGACGTTGATATGGCA |
| mDll1 | ACTCCTTCAGCCTGCCTGA | TATCGGATGCACTCATCGC |
| hDll1 | TCCTGATGACCTCGCAACAGA | ACACACGAAGCGGTAGGAGT |
| mDll3 | CTGGTGTCTTCGAGCTACA | ACACGTGCTAGCAGGTTCC |
| hDll3 | CACTCCCGGATGCACTCAAC | CCCGAGCGTAGATGGAAGGA |
| hDll4 | TCCAACTGCCCTTCAATTTCAC | CTGGATGGCGATCTTGCTGA |
| Nanog | ATGAAGTGCAAGCGGTGGCAGAAA | CCTGGTGGAGTCACAGAGTAGTTC |
| Oct4 | GGCGTTCTCTTTGGAAAGGTGTTC | CTCGAACCACATCCTTCTCT |
| Rex1 | AGGCCAGTCCAGAATACCAG | GGAACTCGCTTCCAGAACCT |
| FGF5 | ACCCTTTGAGCTTTCTACCC | CCGTCTGTGGTTTCTGTTGAGG |
| BLBP | GGGTAAGACCCGAGTTCCTC | ATCACCACTTTGCCACCTTC |
| T-brachyury | GTGACTGCCTACCAGAATGA | ATTGTCCGCATAGGTTGGAG |
| Paraxis | CATCTGCACCTTCTGTCTCA | TCCTTACACAACGCAGGAGT |
| PDGFRa | TGGAGGACAGCTTCCTGTAA | TGCTGGAGTTGTCTGCAGTA |
| HNF3beta | CCCTACGCCAACATGAACTCG | GTTCTGCCGGTAGAAAGGGA |
| GATA6 | GCAATGCATGCGGTCTCTAC | CTCTTGGTAGCACCAGCTCA |
| GATA4 | TCGTAATGCCGAGGGTGAGC | TGGCCTGCGATGTCTGAGTG |
| Sox 2 | GGCGGCAACCAGAAGAACAG | GCTTGGCCTCGTCGATGAAC |
